# JACKIE: Fast Enumeration of Genome-Wide Single- and Multicopy CRISPR Target Sites and Their Off-Target Numbers

**DOI:** 10.1089/crispr.2022.0042

**Published:** 2022-08-12

**Authors:** Jacqueline Jufen Zhu, Albert Wu Cheng

**Affiliations:** ^1^ School of Biological and Health Systems Engineering, Arizona State University, Tempe, Arizona, USA; University of Connecticut Health Center, Farmington, Connecticut, USA.; ^2^ The Jackson Laboratory for Genomic Medicine, Farmington, Connecticut, USA; University of Connecticut Health Center, Farmington, Connecticut, USA.; ^3^ The Jackson Laboratory Cancer Center, Bar Harbor, Maine, USA; University of Connecticut Health Center, Farmington, Connecticut, USA.; ^4^ Department of Genetics and Genome Sciences, University of Connecticut Health Center, Farmington, Connecticut, USA; and University of Connecticut Health Center, Farmington, Connecticut, USA.; ^5^ Institute for Systems Genomics, University of Connecticut Health Center, Farmington, Connecticut, USA.

## Abstract

Zinc finger protein-, transcription activator like effector-, and CRISPR-based methods for genome and epigenome editing and imaging have provided powerful tools to investigate functions of genomes. Targeting sequence design is vital to the success of these experiments. Although existing design software mainly focus on designing target sequence for specific elements, we report here the implementation of Jackie and Albert's Comprehensive K-mer Instances Enumerator (JACKIE), a suite of software for enumerating all single- and multicopy sites in the genome that can be incorporated for genome-scale designs as well as loaded onto genome browsers alongside other tracks for convenient web-based graphic-user-interface-enabled design. We also implement fast algorithms to identify sequence neighborhoods or off-target counts of targeting sequences so that designs with low probability of off-target can be identified among millions of design sequences in reasonable time. We demonstrate the application of JACKIE-designed CRISPR site clusters for genome imaging.

## Introduction

Zinc finger protein (ZFP), transcription activator like effector (TALE), and CRISPR-Cas systems have revolutionized genome biology and synthetic biology by providing versatile DNA and RNA-targeting modalities for engineering.^1–3^ These technologies have revolutionized genome research by providing versatile tools for “writing” the genome, epigenome, and transcriptome as well as “reading” the dynamics of the genome architecture through live-cell genome imaging. The success of these experiments depends heavily on the design of target sequences within the genome.

Although genome and epigenome editing experiments usually require target sequences that occur only once in the genome, genome imaging and loop engineering experiments require one-copy sites or clustered sequences depending on the specific method used and the targets of interest.^4–17^ Most of the existing design software packages provide small scale or one-by-one design.^18–20^ For large-scale design, these software packages are inefficient. Furthermore, no design software is available for genome imaging or loop engineering experiments requiring clustered repetitive sequences.^4,5,7,10,12–14,17^

For enumeration of all potential target sites of length k (k-mers) and their locations in the genome, we implemented Jackie and Albert's Comprehensive K-mer Instances Enumerator (JACKIE) software package that is compatible with high performance computing (HPC) clusters. This allows the generation of databases of single- or multicopy target sites that can not only be used in genome-wide library design but can also be loaded as genome tracks on genome browsers,^21^ alongside other genomic tracks to allow for graphic-user-interface (GUI)-enabled design of targeting experiments.

We generated databases of CRISPR target sites (JACKIEdb) in human hg38 and mouse mm10 genomes. To demonstrate a genome-wide design use case, we queried JACKIEdb to design guide RNAs (gRNAs) for all gene promoters and active enhancers. We also selected several clustered repetitive gRNAs and performed genome imaging experiments.

## Materials and Methods

### Implementation of JACKIE

**FIG. 1.**
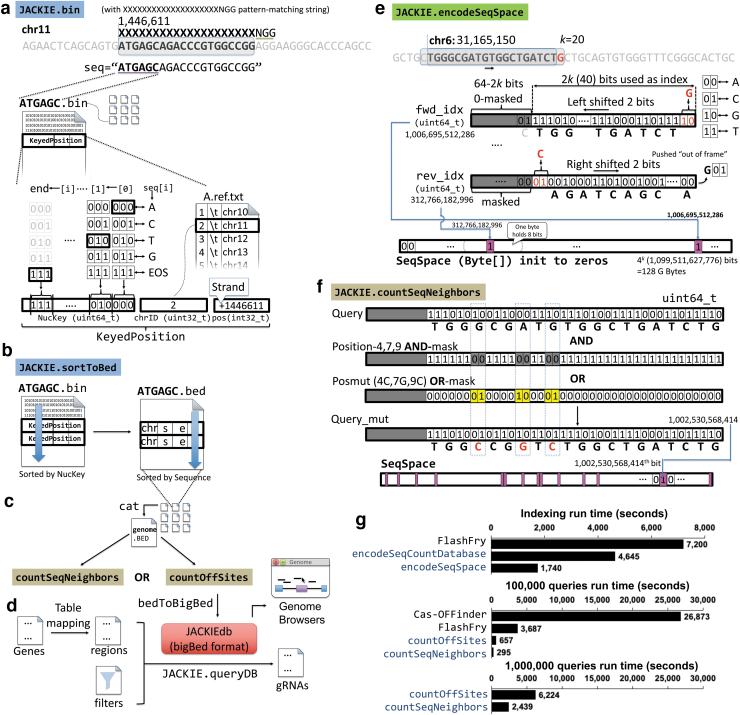
Implementation and evaluation of JACKIE. **(a)** JACKIE.bin scans the genome for k-mers matching a specified motif (e.g., XXXXXXXXXXXXXXXXXXXXNGG matches 20-mer denoted by 20X followed by NGG PAM required by the CRISPR/SpCas9 system), and encodes each occurrence in a data structure called KeyedPosition, consisting of NucKey (unsigned 64-bit integer, uint64_t), chrID (unsigned 32-bit integer, uint32_t), and pos (signed 32-bit integer, int32_t). NucKey records a binary representation of the k-mer sequence using three bits per nucleotide. chrID records an integer representing a chromosome. A chrID-chromosome mapping file is generated. pos records the location of the binding site, with negative and positive integers representing the minus strand and positive strand, respectively. JACKIE.bin outputs KeyedPosition to files as it scans the genome. To parallelize this step, four processes are started as separate cluster jobs, each focusing on the A, C, T, or G as the first nucleotide of k-mer. To allow for parallelization in subsequent steps, KeyedPosition records are appended to files per 6-mer prefix of the k-mer (<6merPrefix>.bin, e.g., ATGAGC.bin contains all records for all k-mer starting with ATGAGC). **(b)** JACKIE.sortToBed loads each <6merPrefix>.bin file and performs a sort on the KeyedPosition records on the NucKey variable and then traverses the sorted list of KeyedPosition to output a bed interval file containing the coordinate position of each k-mer site with item named by <NucKey>.<copy number>/<sequence>, and score field recording the copy number. Parallelization is achieved by either starting separate jobs on individual <6merPrefix>.bin files or by starting batch jobs on 2mer prefixes (e.g., AA AT AC AG operating on AA*.bin, AT*.bin, AC*.bin, AG*.bin, etc.). **(c)** The outputs (<6merPrefix>.bed) are then merged into a combined bed file using cat Unix command. The combined bed file is then subjected to off-target analysis using JACKIE.countSeqNeighbors or JACKIE.countOffSites. **(d)** bedToBigBed is then used to convert bed files to binary bigBed formatted JACKIEdb for fast downstream queries and visualization on genome browsers. JACKIE.queryDB takes in regions of interest and filters as well as sorting criteria to query JACKIEdb to select gRNAs. **(e)** Evaluation of off-target effects starts with generating a bit array representation of sequence space (SeqSpace) of the target genome. JACKIE.encodeSeqSpace initializes a 4^*k*^-bit (i.e., 4^2*k*-3^ bytes) array to 0's as an “empty” SeqSpace. The program scans the genome and records the presence of each encountered k-mer by first translating the sequence to a binary index, and then setting the bit on the SeqSpace referenced by the index to 1. The reverse-complement k-mer is handled similarly. A sliding mechanism that shifts two bits as the genome advances to the next nucleotide is used for both indices for time efficiency. **(f)** JACKIE.countSeqNeighbors reads in SeqSpace and a table of query sequences (e.g., target site design bed files from JACKIE.sortToBed) and computes the number of sequence neighbors with different number of mismatches up to a specified threshold. At start, JACKIE.countSeqNeighbors builds a collection of position AND-masks and pos_mut OR-masks that when applied to a query sequence encoded in the binary representation generate all possible neighbor sequences up to the specified mismatch number. The resultant query_mut values then serve as the indices to query the SeqSpace array. If the corresponding bit of the array is 1, the sequence neighbor count for that particular mismatch number is incremented by 1. After going through all pairs of AND-mask and OR-mask, the sequence neighbor counts for each mismatch number up to the specified threshold are reported for that particular query sequence. **(g)** Run time comparison for off-target analysis of JACKIE through countSeqNeighbor approach or countOffSites approaches in comparison with FlashFry and Cas-OFFinder in terms of the one-time indexing (*top panel*) and off-target analysis of 100,000 (middle panel) and 1,000,000 (bottom panel) gRNA sequences up to three mismatches. Cas-OFFinder and FlashFry were excluded from the 1,000,000 analyses because we were unable to run them on 1,000,000 gRNA sequences in reasonable time (Cas-OFFinder) and due to an out-of-memory (FlashFry) issue. gRNA, guide RNA; JACKIE, Jackie and Albert's Comprehensive K-mer Instances Enumerator; PAM, proto-adjacent motif.

JACKIE consists of four sets of programs implemented in C++, JACKIE.bin, JACKIE.sortToBed, (JACKIE.encodeSeqSpace, JACKIE.encodeSeqSpaceNGG, JACKIE.encodeSeqSpace.prefixed, JACKIE.encodeSeqCountDatabase), and (JACKIE.countSeqNeighbors, JACKIE.countSeqNeighbors.pmulti, JACKIE.countOffSites); Python and Bash scripts for job batching and downstream processing; as well as JACKIE.queryDB implemented in C that allows fast filtering and extraction of gRNAs within regions of interest from gRNA databases (JACKIEdb) generated by JACKIE (Fig. 1). JACKIE.bin scans the genome and enumerates any potential targeting sites (k-mers) on both strands (Fig. 1a).

A pattern-matching string parameter allows the specification of length k of the k-mer binding sites, as well as motif constraints (such as NGG in the case of CRISPR-SpCas9). Although in this article we focus on CRISPR-SpCas9 designs, the pattern-matching string parameter allows enumeration of target sites for other CRISPR orthologs, ZFPs, and TALEs. To enhance memory and computation efficiency, k-mers are mapped to an unsigned 64-bit integer (uint64_t NucKey) by encoding each nucleotide and an end-of-string (EOS) indicator in 3 bits, A->000, C->001, T->010, G->011, and EOS->111 (Fig. 1a).

The chromosome (unsigned 32-bit integer, uint32_t chrID) and location for each sequence instance (signed 32-bit integer, int32_t pos) are recorded such that chrID maps to a chromosome through a tab-delimited table while the sign of pos registers the positive (+) and negative (−) strands. The aggregate KeyedPosition data (NucKey, ChrID, pos) generated at each position during the genome scan are written into files prefixed by a predefined prefix length (default to 6, e.g., ATGAGC.bin contains all KeyedPosition of k-mers prefixed by ATGAGC) so that the next steps can be highly parallelized.

The JACKIE.bin operations can be divided into four subtasks each working on k-mers prefixed by each of the four bases (e.g., the “A” subtask generates all AXXXXX.bin files and an A.ref.txt chromosome table). Upon the completion of the JACKIE.bin operations, JACKIE.sortToBed is run on each bin file by loading the KeyedPosition records into a list in memory, and sorting them by NucKey, thus placing KeyedPosition records of identical k-mers adjacent to each other in the list (Fig. 1b). The NucKey-sorted list is then traversed to output k-mer sites in bed files,^22^ which can be concatenated to generate a genome-wide target bed file.

The genome-wide bed file can be subjected next to off-target analysis through JACKIE.countSeqNeigbhors or JACKIE.countOffSites (Fig. 1c) and ultimately converted into JACKIEdb encoded in bigBed format,^22^ which can be uploaded to University of California, Santa Cruz (UCSC) genome browsers for GUI-based visualization or rapid query using JACKIE.queryDB (Fig. 1d). The extensive divide-and-conquer design and binary encoding of sequences allow JACKIE to sort sequences efficiently and be highly parallelized on an HPC cluster.

Off-target effects of engineered nucleases happen when nontarget sites with similar sequences to the target sites are recognized and unwanted effects are induced. To prevent or reduce off-target effects, the simplest method is to filter for target sites with fewer predicted off-target locations having the same or similar sequences. One of the most popular software for CRISPR off-target predictions is Cas-OFFinder, which enumerates sequences and their occurrences up to a certain number of mismatches to the designed gRNAs.^18^ Although very efficient in identifying off-target sites for a few gRNAs, evaluation of millions of gRNA designs using Cas-OFFinder would be impractical, so we first implemented off-target evaluation strategies in terms of “neighborhood” sequences of the target in the sequence space.

The goal is to identify the number of k-mer sequences (in terms of their presence or absence but not their actual locations nor copy numbers) that are a certain number of mismatches (e.g., 3), or hamming distances, away from the query design sequence. The off-target prediction routines consist of two programs: JACKIE.encodeSeqSpace (or JACKIE.encodeSeqSpaceNGG for CRISPR-SpCas9-PAM constrained sequence subspace) for “indexing” and JACKIE.countSeqNeighbors for actual queries (Fig. 1e, f). JACKIE.encodeSeqSpace scans genome sequence files and records the presence or absence of each possible k-mer in a bit array representation (“index”) of the k-mer sequence space (SeqSpace) (Fig. 1e).

At the beginning, SeqSpace is sized at 4^*k*^ bits, that is, 4^*k*^/8 = 2^2*k*-3^ bytes, and initialized with 0's. As the genome sequence is scanned, each encountered k-mer sequence is mapped per nucleotide to 2-bit representation (A →00, C → 01, G → 10, T → 11), and packed into an unsigned 64-bit integer (uint64_t). The unused most significant bits are zero-masked. The forward strand sequence is mapped to the fwd_idx variable, whereas the reverse complementary sequence is mapped to the rev_idx variable. To ensure computational efficiency, a “sliding” scheme is used such that the forward index (fwd_idx) is shifted left by two bits whereas the reverse complement index (rev_idx) is shifted right by two bits as the scanning process advances to the next nucleotide, and two bits for the new encountered nucleotide (or the reverse complement) are then added to the least or most significant bit positions, respectively, for fwd_idx and rev_idx.

The bits of SeqSpace at the positions indexed by fwd_idx and rev_idx are then set to 1, recording the presence of the k-mer and its reverse complement in the SeqSpace. At completion, SeqSpace will have recorded in each bit the presence (bit value 1) or absence (bit value 0) of a corresponding indexed k-mer in the genome. The SeqSpace array will then be outputted to disk as compressed seqbits.gz to allow for future uses. JACKIE.countSeqNeighbors loads SeqSpace from the seqbits.gz file and a list of target site sequences, then counts the number of sequence neighbors each target site sequence has for each number of mismatches up to a user-provided threshold (e.g., 3) (Fig. 1f).

The binary representation of k-mers and the indexed SeqSpace allow fast computation of sequence neighbors. JACKIE.countSeqNeighbors first generates all position bitwise AND-masks and OR-masks that allow all permutation of sequence changes of a query k-mer through a bitwise AND and a bitwise OR operation, respectively. An example pair of AND- and OR-masks for changing three bases of the query in the binary representation is shown in Figure 1e. For each query sequence, JACKIE.countSeqNeighbors applies the AND- and OR-mask pairs one-by-one, and looks up the bit indexed by the resultant Query_mut variable in the SeqSpace array, incrementing the count of sequence neighbors for the corresponding number of mismatches by 1.

After completing all searches for the query sequence, the number of sequence neighbors is outputted per mismatch number. For computers that do not support 128GB memory for 20-mer SeqSpace, we implemented “divide-and-conquer” versions, JACKIE.encodeSeqSpace.prefixed and JACKIE.countSeqNeighbors.pmulti that divide the problems into prefixed subspaces. For example, using 2 nucleotide (nt) prefix, we divided the problem into 16 subspaces (AA, AC, AG, AT, …), each requiring only 8 GB memory for *k* = 20. If needed, the programs allow further division of subspaces.

For users interested to obtain the exact off-target site counts, we implemented JACKIE.encodeSeqCountDatabase and JACKIE.countOffSites that encode SeqSpace with exact copy numbers and report exact off-target site counts, with the off-target sequences with mismatch positions optionally marked by lowercase letters, respectively. A user-specified number of bits (*b*) is used to record the copy number of a particular k-mer in the genome up to 2^*b*^-2 copies in the SeqSpace. If the copy number exceeds 2^*b*^-2, the SeqSpace record is set to 2^*b*^-1, and the copy number is recorded in a “overflow” red-black tree in the C++ Standard Template Library map class, by setting the key to the two-bit representation of the k-mer (uint64_t) and the value to the copy number.

For fast query of the gRNA databases generated by JACKIE, we encoded one-copy gRNA JACKIEdb and multicopy (> = 2 copies) gRNA JACKIEdb in bigBed format with the following AutoSql structures:

One-copy JACKIEdb:

table OneCopyPAMOffSiteCounts
“One-copy CRISPR SpCas9 NGG sites and off-target counts up to 3 mismatches”
(
string chrom;
uint chromStart;
uint chromEnd;
string name;
uint score;
char[1] strand;
uint thickStart;
uint thickEnd;
uint reserved;
uint n0mismatches; “Number of exact matched sites”
uint n1mismatches; “Number of 1-mismatch sites”
uint n2mismatches; “Number of 2-mismatch sites”
uint n3mismatches; “Number of 3-mismatch sites”
uint totalOffSites; “Total number of 1,2,3-mismatch sites”
string offSiteCounts; “String representation of offsite counts separated by slashes”
string spacerSeq; “Spacer sequence of gRNA”
uint percentGC; “Percent GC of spacer sequence”
uint longestTandemT; “Longest run of T”
)

Multicopy JACKIEdb:

table TwoPlusCopyPAMOffSiteCounts
“One-copy CRISPR SpCas9 NGG sites and off-target counts up to 3 mismatches”
(
string chrom;
uint chromStart;
uint chromEnd;
string name;
uint score;
char[1] strand;
uint thickStart;
uint thickEnd;
uint reserved;
int blockCount; “Number of blocks”
int[blockCount] blockSizes; “Comma separated list of block sizes”
int[blockCount] chromStarts; “Start positions relative to chromStart”
uint clusterSize; “Size of gRNA cluster”
uint n0mismatches; “Number of exact matched sites”
uint n1mismatches; “Number of 1-mismatch sites”
uint n2mismatches; “Number of 2-mismatch sites”
uint n3mismatches; “Number of 3-mismatch sites”
uint totalOffSites; “Total number of 1,2,3-mismatch sites”
string offSiteCounts; “String representation of offsite counts separated by slashes”
string spacerSeq; “Spacer sequence of gRNA”
uint percentGC; “Percent GC of spacer sequence”
uint longestTandemT; “Longest run of T”
)

To enable rapid query of JACKIEdb, we modified Kent's code for bigBedToBed^22^ to create JACKIE.queryDB that accepts a list of regions and a list of filters as well as sorting criteria to extract matching target sequences records from bigBed-encoded JACKIEdb. JACKIE.queryDB loads regions of interest, filters, and sorting criteria into memory as lists or arrays. As the chromosome records in the JACKIEdb bigBed file is looped through, if the chromosome is present in the regions list, JACKIE.queryDB dives into the chromosome record and requests target sites in each region.

The returned target sites are then passed through the list of filters. If the “BEST *n*” option is selected to output *n* best target sequences according to a series of sorting criteria, an array of size *n* is created to hold the updated list of BEST *n* target sequences encountered during the traversal. JACKIE.queryDB then outputs the BEST *n* or all gRNAs passing through the filters in the specified regions in extended bed file with columns as described in the AutoSql structures mentioned.

### Analysis of hg38 JACKIEdb

To analyze the distribution of gRNAs, we divided each chromosome into one-megabase bins, and used JACKIE.queryDB to extract gRNAs within each bin's matching defined criteria and counted the number of sites or clusters in each bin. For bins with <1 Mb length at the ends of chromosomes, the site or cluster numbers were normalized to the 1 Mb length. The site or cluster numbers were then plotted per bin using R (https://www.R-project.org/).

### CRISPR imaging experiments

gRNA spacer sequences were cloned into gRNA-15xPBSc expression vector through an oligo-annealing protocol as previously described.^6^ HEK293T cells were cultivated in Dulbecco's modified Eagle's medium (Sigma) with 10% fetal bovine serum (FBS)(Lonza), 4% Glutamax (Gibco), 1% sodium pyruvate (Gibco), and penicillin–streptomycin (Gibco). Incubator conditions were 37°C and 5% CO_2_. Cells were seeded into 24-well plates the day before being transfected with 50 ng pAC1445-dCas9 plasmid (Addgene #73169), 25 ng pAC1447-Clover-PUFc plasmid (Addgene #73689) and 250 ng sgRNA-15xPBSc plasmid. Cells were fixed, mounted on slides, and imaged 48 h after transfection on a Leica SP8 confocal microscope.

## Results

We ran JACKIE.bin (4 jobs) and JACKIE.sortToBed (16 jobs) on a Unix HPC cluster with Intel Xeon compute cores for hg38 human and mm10 mouse genomes, which took a total of ∼35 and ∼22 min, respectively. On a MacBook Pro laptop (2021; MacBookPro18,3; 14-inch; Apple M1 Pro chip; 16GB ram; 1TB SSD; MacOS 12.2.1 Monterey; hereafter refer to as MacBookPro), running JACKIE.bin on hg38 human genome with 4 processes took ∼12 min, and JACKIE.sortToBed with 16 processes took ∼27 min. Thus, JACKIE.bin and JACKIE.sortToBed ran on the MacBookPro for a total of ∼40 min. JACKIE.encodeSeqSpaceNGG on hg38 took 45 min (25 min spent on encoding, 20 min spent on compressing to seqbits.gz file) on one node of the Unix HPC cluster.

We ran JACKIE.countSeqNeighbors on 52,666,035 single-copy CRISPR query sequences for up to three mismatches that took 12.5 h on one node of the Unix HPC cluster. On the MacBookPro, running JACKIE.encodeSeqCountDatabase on hg38 with three-bit SeqSpace took 4645 s (∼1.3 h) total when 16 runs of 2-nt subspaces (*k* = 20) were executed in series. On the same machine, running JACKIE.encodeSeqSpace.prefixed took a total of 29 min. Sequence Count Database or SeqSpace encoding (“indexing”) is only needed once for a particular genome for a particular k and PAM. We compared speeds of the off-target prediction portion of JACKIE with Cas-OFFinder^18^ and FlashFry^23^ (Fig. 1g). Cas-OFFinder is one of the first and most popular off-target prediction software whereas FlashFry was evaluated to be the fastest among existing off-target prediction software packages by a previous article.^24^ Indexing of FlashFry database took 2 h. Running Cas-OFFinder, FlashFry, JACKIE.countOffSites, and JACKIE.countSeqNeighbors.pmulti on 100,000 queries took 26,873 s (∼7.5 h), 3687 s (∼1 h), 657 s (∼11 min), and 295 s (∼5 min), respectively. We ran JACKIE.countOffSites and JACKIE.countSeqNeighbors.pmulti on 1,000,000 queries that took 6224 s (∼1.72 h) and 2439 s (∼40 min), respectively.

We were unable to run Cas-OFFinder on 1,000,000 sequences within reasonable time while FlashFry failed to run on 1,000,000 with the memory constraint of our computers. Note these are not completely fair comparisons as Cas-OFFinder reports the locations of off-target sites allowing DNA/RNA bulges in addition to mismatches, whereas FlashFry and JACKIE.countOffSites report the number of off-target sites only. The fastest approach taken by JACKIE.countSeqNeighbors.pmulti reports the number of neighborhood sequences only. For large-scale target design projects only requiring target sites with few or no off-targets up to a certain mismatch threshold, off-target prediction approaches taken by JACKIE could suffice and provide time-effective solutions.

**FIG. 2.**
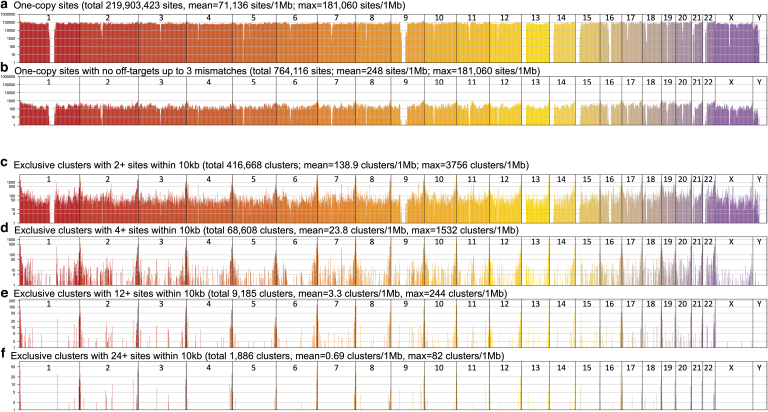
Distributions of one-copy gRNA sites or multicopy gRNA clusters in the human genome. **(a)** Column plot showing the number of one-copy sites (*y*-axis) across the human genome in megabase bins (*x*-axis). Chromosome numbers are indicated on *top*. **(b)** Column plot showing the number of one-copy sites with no off-targets up to three mismatches across the human genome in megabase bins. **(c)** Column plot showing the number of multicopy gRNA site clusters with two or more sites across the human genome in megabase bins. **(d)** Column plot showing the number of multicopy gRNA site clusters with four or more sites across the human genome in megabase bins. **(e)** Column plot showing the number of multicopy gRNA site clusters with 12 or more sites across the human genome in megabase bins. **(f)** Column plot showing the number of multicopy gRNA site clusters with 24 or more sites across the human genome in megabase bins.

To allow the genome browser to handle billions of gRNA designs as well as for rapid downstream filtering, we converted bed files generated by JACKIE to bigBed format,^22^ and implemented JACKIE.queryDB for quick extraction of user-specified number of optimal gRNAs within a list of genomic regions and satisfying user-defined filtering and sorting criteria on gRNA properties and off-target profiles. Using JACKIE.queryDB, we analyzed the distribution of one-copy gRNA sites or multicopy gRNA clusters across the human genome in megabase bins (Fig. 2). There are ∼220 million one-copy gRNA sites in the human genome, with an average density of 71,136 sites per megabase bin (Fig. 2a). After filtering for the absence of off-target sites up to 3 mismatches, there are 764,116 sites remaining, with an average of 248 sites in each megabase bin (Fig. 2b). We also evaluated the availability of multicopy gRNA site clusters. We considered only clusters that have gRNA sites exclusively within a 10 kb window. There are a total of 416,668 clusters within with two or more sites, a density of 138.9 clusters per megabase bin. These numbers decreased as we increased the minimal copy number to 4 (68,608 clusters; 23.8 clusters/Mb), 12 (9185 clusters, 3.3 clusters/Mb), and 24 (1886 clusters, 0.69 clusters/Mb).

**FIG. 3.**
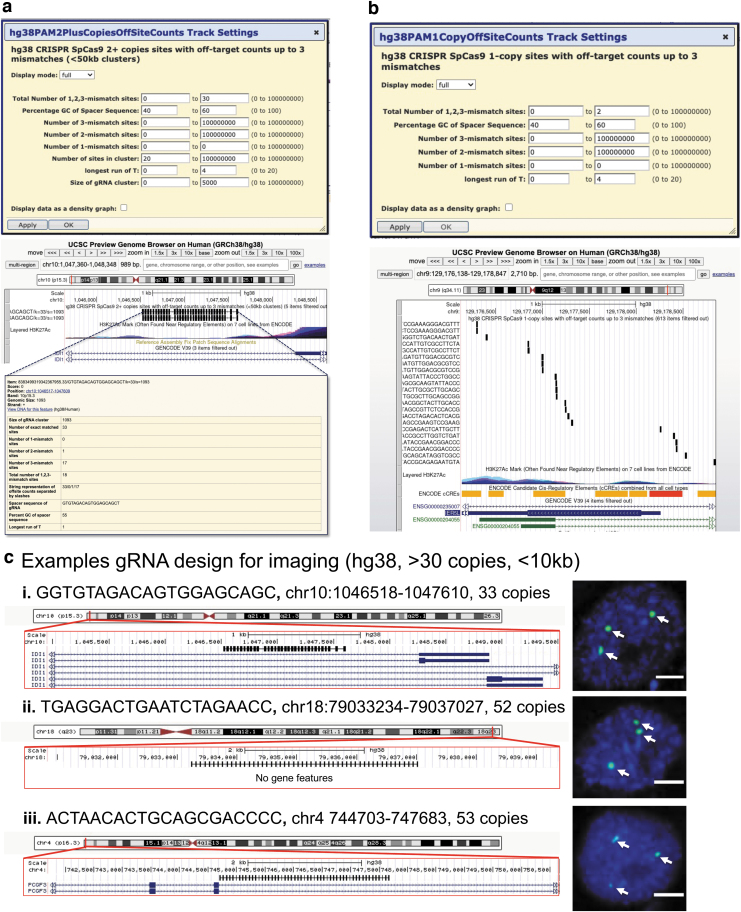
Example use cases for JACKIE designs. **(a)** A screenshot of UCSC genome browser showing CRISPR/SpCas9 gRNA clusters. *Top:* Example track filter settings for 30 or fewer total off-targets up to three mismatches, 40–60%GC spacer, no one mismatch off-targets, 20 or more sites in cluster, spacer not containing more than runs of four or more T, cluster smaller than or equal to 5 kb. *Middle:* Example gRNA clusters after filtering. *Bottom:* Information page of the first cluster shown after clicking on the item. **(b)** Screenshot of UCSC genome browser showing single-copy CRISPR-SpCas9 gRNA sites. *Top:* Example track filter settings for up to two off-target sites, 40–60%GC spacer, no one mismatch off-targets, spacer not containing runs of four or more T. **(c)** To demonstrate genomic imaging use case, we selected three gRNA clusters and performed CRISPR genome imaging experiment. UCSC genome browser screenshots are shown on the left with the line-bar annotating the CRISPR sites, and representative fluorescence microscopy images shown on the right. Microscopy images were derived by merging green (CRISPR) and blue (DAPI: nucleus stain). *Arrows* point to fluorescent foci of interest. Scale bars, 5 μm. DAPI, 4′,6-diamidino-2-phenylindole.

Two-copy clusters can be used with SNP-CLING.^9^ Four-copy clusters can be used with Qin's method.^8^ Imaging methods requiring 12 or more sites^4,5,7,10,11,13^ will only be able to image gross chromosome positions or features using these multicopy gRNA clusters. For nonrepetitive regions, these imaging methods would require the use of a set of multiple one-copy gRNAs (Fig. 2a). Alternatively, one could use one-copy gRNAs in combination with the Casilio-Imaging method requiring only one gRNA per nonrepetitive target.^15,16^

To evaluate the performance and potential of JACKIEdb for designing a genome-wide gRNA library, we used JACKIE.queryDB to design a library of one-copy gRNAs targeting 633,671 ChromHMM active enhancers,^25^ with the goal to identify the 12 best one-copy gRNAs per enhancer sorted by (and minimizing) total off-target counts. The search recovered 7,324,597 gRNAs total in 197 s (3.28 min) on the MacBookPro. Similarly, we conducted a search on 232,674 five-kilobase promoters of UCSC KnownGenes,^21^ recovering 2,545,407 total gRNA designs in 162 s (2.7 min) on the MacBookPro.

For GUI-based design on genome browsers, we generated UCSC genome browser sessions preloaded with human (hg38) and mouse (mm10) JACKIEdb tracks on which users can filter for specific gRNA properties, including the numbers of 1-, 2-, and 3-mismatch sites, total off-target site number, percentage GC (GC%) of spacer sequence, longest run of T as well as number of sites and size of the gRNA clusters (Fig. 2a,b). Not only can users filter for desired gRNA properties, but they can also select gRNAs alongside hundreds of tracks already available on the UCSC genome browser. We selected three CRISPR binding clusters and applied CRISPR imaging^6^ to visualize them through fluorescence microscopy (Fig. 2c), producing three fluorescent foci in each case, consistent with the ploidy of the HEK293T cells used in the experiments.

## Discussion

Advances in engineered nuclease technologies have fueled an explosion of genome, epigenome, and transcriptome editing and imaging tools. The easily programmable feature, as exemplified by the CRISPR systems, allows large-scale genome-wide experiments. Although some applications, such as genome and epigenome editing, require target sites that are unique, that is, with sequence occurring only once in the genome, some imaging methods require multiple binding sites that are clustered within a particular region. Existing target design software packages focus on individual designs but are inefficient in large-scale design. In this article, we described a new software package, JACKIE, that enumerates all single- and multicopy k-mers in a target genome as well as provides fast evaluation of off-target effects.

The off-target prediction algorithm is nearly 100-fold more time efficient than the most popular software for off-target prediction, making the off-target prediction of millions of sequences practical. Genome-wide gRNA databases generated by JACKIE (JACKIEdb) and encoded in bigBed format can be conveniently loaded onto GUI-enabled genome browsers alongside other tracks so that users can design target sequences with respect to annotated elements or epigenomic features. We computed CRISPR-SpCas9 single-copy and multicopy JACKIEdb for human hg38 and mouse mm10 genomes that can be directly downloaded or visualized on UCSC genome browser.

We implemented JACKIE.queryDB for rapid extraction of gRNAs from JACKIEdb within genomic regions of interest sorted by and matching defined criteria. We used JACKIE.queryDB to generate gRNA libraries targeting human gene promoters and active enhancers. As a demonstration for the utility of clustered repetitive gRNA designed by JACKIE, we selected three <10 kb clusters of >30 gRNAs and performed CRISPR genome imaging. We believe that JACKIE and JACKIEdb will facilitate target designs for different CRISPR applications including large-scale or genome-wide gRNA design projects.

## Data Availability

Source codes and JACKIEdb for hg38 and mm10 are available for download at (https://cheng.bio/JACKIE/ and https://github.com/albertwcheng/JACKIE2). A preprint of this article is available on BioRxiv at (https://doi.org/10.1101/2020.02.27.968933).
